# Concurrent Optical Gain Optimization and Electrical Tuning in Novel Oligomer:Polymer Blends with Yellow‐Green Laser Emission

**DOI:** 10.1002/advs.201801455

**Published:** 2018-11-08

**Authors:** Qi Zhang, Qi Wei, Xiangru Guo, Gang Hai, Huizhi Sun, Jiewei Li, Ruidong Xia, Yan Qian, Santiago Casado, José Raúl Castro‐Smirnov, Juan Cabanillas‐Gonzalez

**Affiliations:** ^1^ Key Laboratory for Organic Electronics and Information Displays (KLOEID) Jiangsu‐Singapore Joint Research Center for Organic/Bio Electronics and Information Displays Institute of Advanced Materials (IAM) Nanjing University of Posts and Telecommunications 9 Wenyuan Road Nanjing 210046 P. R. China; ^2^ IMDEA Nanoscience Calle Faraday 9 Cantoblanco 28049 Madrid Spain; ^3^ Shaanxi Institute of Flexible Electronics (SIFE) Northwestern Polytechnical University (NPU) 127 West Youyi Road Xi'an 710072 Shanxi China; ^4^ Key Laboratory of Flexible Electronics (KLOFE) and Institute of Advanced Materials (IAM) Jiangsu National Synergistic Innovation Center for Advanced Materials (SICAM) Nanjing Tech University (NanjingTech) 30 South Puzhu Road Nanjing 211816 China

**Keywords:** distributed feedback lasers, Förster resonance energy transfer, oligofluorenes, organic lasers, organic semiconductors

## Abstract

Electrically pumped organic lasing requires the integration of electrodes contact into the laser cavity in an organic light‐emitting diode (OLED) or organic field effect transistor configuration to enable charge injection. Efficient and balanced carrier injection requires in turn alignment of the energy levels of the organic active layers with the Fermi levels of the cathode and anode. This can be achieved through chemical substitution with specific aromatic functional groups, although paying the price for a substantial (and often detrimental) change in the emission and light amplifying properties of the organic gain medium. Here, using host–guest energy transfer mixtures with hosts bearing a systematic and gradual shift in molecular orbitals is proposed, which reduces the amplified spontaneous emission (ASE) threshold of the organic gain medium significantly while leaving the peak emission unaffected. By virtue of the low guest doping required for complete host‐to‐guest energy transfer, the injection levels in the blends are attributed to the host whereas the gain properties solely depend on the guest. It is demonstrated that the ASE peak and thresholds of blends with different hosts do not differ while the current efficiency of OLEDs devices is deeply influenced by molecular orbital tuning of the hosts.

Conjugated polymers (CP) have received continuous attention in the last two decades as laser gain medium owing to their solution processability, high photoluminescence quantum efficiencies (PLQE), large stimulated emission cross sections, and chemically tunable emission wavelengths.^1^, ^2^, ^3^, ^4^ CP lasing has up to now only been achieved upon optical pumping^5^, ^6^, ^7^ while electrically pumped laser action still remains a major challenge.^8^ However, this long standing goal might become surmountable in a short term in view of the recent developments on quasi‐continuous wave optically pumped lasing^9^ and the recent demonstration of low threshold population inversion‐free organic polariton lasers.^10^, ^11^

Among the several milestones to pave the road toward organic electrical pumping an important aspect is the concurrency of outstanding charge transport, emission, and optical gain properties. Tuning of the CP electronic properties while leaving unaltered the capability of the CP active layer to amplify light is however not straightforward. The highest occupied and lowest unoccupied molecular orbitals (HOMO and LUMO, respectively) of CPs can be, for instance, tailored upon backbone functionalization with a variety of moieties.^12^, ^13^ Fluorene‐based copolymers constitute a paradigmatic example wherein, starting with unsubstituted poly(9,9‐dioctyl fluorene), the HOMO and LUMO levels are pushed inside the bandgap by a 0.5–1 eV range upon attachment of electron donating and/or withdrawing groups to the fluorene monomer, concomitant with a redshift of the photoluminescence (PL).^14^, ^15^, ^16^, ^17^ The use of these copolymers as active layers on organic light‐emitting diodes (OLEDs) has proved to provide better charge transport balance,^18^ increase charge recombination,^19^ and efficient blue‐to‐red tuning of electroluminescence (EL).^20^, ^21^, ^22^, ^23^, ^24^, ^25^ Despite the validity of this approach for OLEDs, the optical gain properties of fluorene‐based copolymers deteriorate as the PL, and consequently the stimulated emission, is shifted from blue to red. For instance, the net optical gain coefficient of PFO is ≈74 cm^−1^, while those of poly(9,9‐dioctylfluorene‐*co*‐benzothiadiazole) F8BT and Red F are only 22 and 24 cm^−1^, respectively.^26^ Shifting the emission to longer wavelengths leads to an increased spectral overlap between stimulated emission and excited‐state absorption,^27^ triggering exciton–exciton annihilation processes due to exciton diffusion and enhanced exciton–exciton Förster radius in low bandgap CPs.^28^, ^29^ Therefore, alternative strategies are required in order to manipulate independently molecular orbitals and emission properties while leaving unaltered optical gain properties of the gain medium material.

In this work, we propose a new approach which combines the use of Förster resonance energy transfer (FRET) and independent tuning of host HOMO/LUMO levels in conjugated host:guest mixtures. We demonstrate that this method allows for tuning of the molecular orbitals leaving unaltered the PL and ASE spectra as well as the pump excitation thresholds for ASE in the blends. For this purpose we introduce three novel largely steric oligomer hosts with various side substituents, 9‐octyl‐3,6‐bis(2,7,9‐triphenyl‐9H‐fluoren‐9‐yl)‐9H‐carbazole (DPHS), 3,6‐bis(2,7‐bis(3,5‐difluorophenyl)‐9‐phenyl‐9H‐fluoren‐9‐yl)‐9‐octyl‐9H‐carbazole (DF), and 3,6‐bis(2,7‐bis(3,5‐bis(trifluoromethyl)phenyl)‐9‐phenyl‐9H‐fluoren‐9‐yl)‐9‐octyl‐9H‐carbazole (DCF3). The chemical structures and detailed synthesis procedures are provided in the insets of **Figure** **1**a and in the Supporting Information, respectively. Cyclic voltammograms (Figure S1, Supporting Information) demonstrate that fluorination induces an increase in electron affinity and ionization potential values of 0.35 and 0.2 eV owing to the high fluorine electronegativity and the strong dipole moment associated to the C—F bond.^30^, ^31^ These oligomers all share similar properties regardless their side substituents: they possess deep‐blue fluorescence, relatively high PLQE, and outstanding thermal stability with *T*_d_ and *T*_g_ temperatures exceeding 380 and 150 °C, respectively (see Figure S2, Supporting Information).^32^ Furthermore, the three compounds are excellent hosts for poly(9,9‐dioctylfluorene‐*co*‐ benzothiadiazole) (F8BT) to achieve green‐yellow polymer lasers through energy transfer as confirmed by a one order of magnitude lower ASE threshold in blends compared with the threshold of pristine F8BT. The PLQE values of DF/F8BT blends, selected for a detailed study, reflect a twofold increase with respect to that of F8BT, highlighting the beneficial effects of host dilution and energy transfer. The large steric hindrance of the oligomer hosts and the homogeneous morphologies exhibited by the blend films are important factors which contribute to enhance F8BT emission in the condensed state, keeping F8BT chromophores far apart to hinder exciton–exciton annihilation. We demonstrate that dispersing low weight fractions of CPs in hosts with different fluorination degree enables independent tuning of the HOMO/LUMO energy levels while keeping the PL spectra and ASE properties of the blends unaffected.

**Figure 1:**
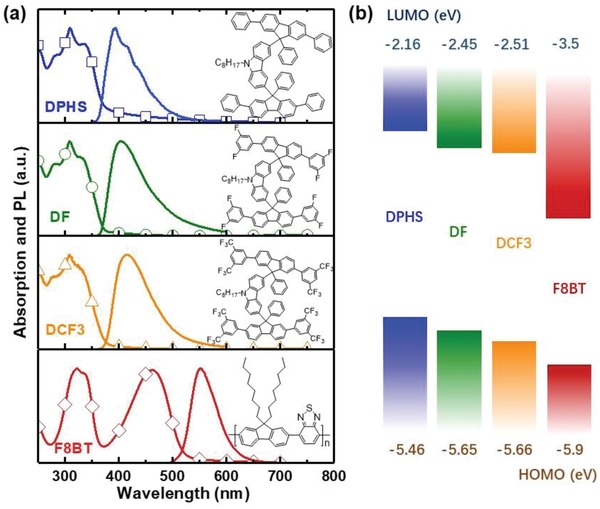
a) Absorption and photoluminescence spectra of (from top to bottom) DPHS, DF, DCF3, and F8BT, together with their chemical structures. b) HOMO and LUMO levels obtained from cyclic voltammetry measurements of DPHS, DF, DCF3, and F8BT.

Figure 1a shows the absorption and PL spectra of three oligomers (DPHS, DF, DCF3) and F8BT. The absorption spectrum of DPHS is characterized by a band with a maximum at 309 nm and a tail extending down to 570 nm. The PL spectrum of DPHS in turn peaks at 394 nm and tails down to 560 nm. The absorption and PL spectra of DF and DCF3 confirm that —F and —CF_3_ fluorination lead to a moderate absorption and PL redshift,^33^, ^34^ with absorption (PL) maxima at 309 (394) nm, 309 (403) nm, and 308 (414) nm for DPHS, DF, and DCF3, respectively. The absorption spectrum of F8BT shows two bands centered at 330 and 450 nm, whereas it displays a low absorption valley between 350 and 400 nm. The HOMO and LUMO levels of each oligomer obtained with cyclic voltammetry measurements are shown in Figure 1b. DF exhibits a shift in LUMO (HOMO) energy levels of 0.3 eV (0.2 eV) with respect to the energy level of DPHS. Upon increasing the number of fluorine atoms in the substituents (DCF3), the LUMO (HOMO) level further lowers by 0.06 eV (0.01 eV). The effect of fluorination on the molecular orbitals was further elucidated with density functional theory (DFT) calculations in DPHS, DF, and DCF3 using Gaussian 03 (B3LYP nonlocal density functional with a 6‐31G(d) basis set). The calculated HOMO, LUMO, and electronic wavefunction densities in geometry‐optimized structures are shown in Figure S3 (Supporting Information). The evolution of energy levels upon fluorination follows the same trend on DFT calculations and CV measurements. The key parameters of the three oligomers are shown in Table S1 (Supporting Information).

Next, we explored the luminescent and optical gain properties of the blends composed of fractions of F8BT dispersed in the three host compounds. We monitored the spin‐coated film morphology of the blends of DPHS, DF, and DCF3 with F8BT at 40 wt% F8BT content using atomic force microscopy (AFM). AFM topographies depict highly homogeneous morphologies without evident traces for phase separation in all blends (Figure S4, Supporting Information), indicating large degree of host:guest dispersion. The topographies are rather smooth with typical roughness below 2 nm.

The PL spectra of the three oligomers largely overlap with the F8BT absorption spectrum, all manifesting optimum conditions for resonant energy transfer to F8BT. Absorption and PL spectra of DF/F8BT blends with different F8BT fractions are shown in **Figure** **2**a,b. Starting with pristine DF and increasing the weight fraction of F8BT in blend, the absorption spectra of the blends evolve as a linear spectral superposition of DF and F8BT. Concomitantly, the PL spectra of blends upon 355 nm photoexcitation (predominant host photoexcitation) rapidly switch from the characteristic DF deep blue emission to green‐yellow F8BT fluorescence. The PL from DF component is no longer evident in the blends with more than 20 wt% F8BT, indicating nearly a complete host‐to‐guest energy transfer. Analogous behaviors are observed in DPHS/F8BT and DCF3/F8BT blends (see Figure S5, Supporting Information).

**Figure 2:**
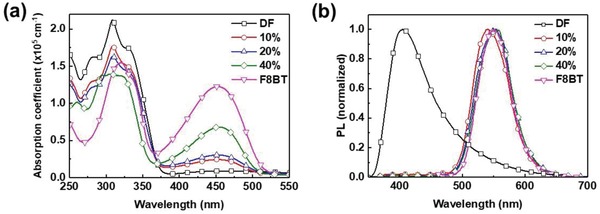
a) Absorption and b) PL spectra of F8BT/DF blend films with different F8BT contents: 0 wt% (squares), 10 wt% (circles), 20 wt% (up‐triangles), 40 wt% (diamonds), and 100 wt% (down‐triangles). The PL spectra were obtained upon 355 nm photoexcitation.

The PLQE values of DF/F8BT blends with different F8BT fractions were obtained (**Figure** **3**a). The PLQE value of pristine F8BT film is about 40%. Dispersing a low weight percentage of F8BT in DF (1 wt% for instance) leads to a substantial PLQE increase up to 74%, concomitant with a 2.5‐fold increase in the PL lifetime (Figure 3a), suggesting the suppression of dynamic quenching processes present otherwise in pristine F8BT.^35^ The PLQE values of blends are well above that of pristine F8BT film. The largest PLQE value (≈78.4%) was measured for 15 wt% F8BT blend with a corresponding PL lifetime of 2.42 ns. Above this concentration the PLQE and PL lifetime values drop, falling to ≈62.3% and 2.07 ns for 50 wt% blends (Table S2, Supporting Information) due to a combination of saturated FRET rates (energy transfer rate rises rapidly from 2.9 × 10^8^ s^−1^ in the 1 wt% F8BT blend to 1.36 × 10^9^ s^−1^ in the 5 wt% F8BT, Figure S6, Supporting Information) and aggregated F8BT chromophores on high F8BT loaded blends, which may play a role on exciton deactivation.

**Figure 3:**
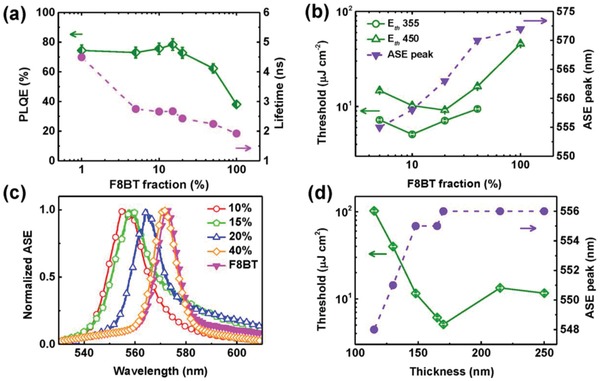
a) Total PLQE of blends (obtained by PL integration across the 400–700 nm range) (diamonds) and fluorescence lifetime values detecting at 560 nm (circles) of F8BT/DF blend films with different F8BT contents photoexcited at 355 and 375 nm, respectively. b) ASE threshold values under 355 nm (circles) and 450 nm (up‐triangles) photoexcitation and ASE peak position (down‐triangles), of F8BT/DF blend films as a function of F8BT fraction. c) Typical ASE spectrum of F8BT/DF blend films with different F8BT contents: 10 wt% (circles), 15 wt% (pentagons), 20 wt% (up‐triangles), 40 wt% (diamonds), and 100 wt% (down‐triangles) obtained upon 355 nm photoexcitation. d) ASE peak position (diamonds) and threshold values (circles) of 10 wt% F8BT/DF film as a function of film thickness under 355 nm photoexcitation.

The optical gain properties of the blends outperform by far those of pristine F8BT regardless the host choice (DPHS, DF, or DCF3). The ASE spectra of F8BT (upon 450 nm photoexcitation) and 40 wt% blends in DPHS, DF, and DCF3 (upon 355 nm photoexcitation) (Figure S7a, Supporting Information) depict narrow peaks at 570.2, 570.1, and 570.6 nm with full width at half maximum (FWHM) values of 11.2, 11.8, and 11.7 nm, respectively. Transition from fluorescence to ASE regimes occurs above a certain pump fluence (cf. ASE threshold) accompanied by a sudden collapse of the emission linewidth (Figure S7b, Supporting Information). Remarkably, the ASE threshold values of 40 wt% blends are very similar regardless the host choice, that is, 0.202 µJ pulse^−1^ (9.21 µJ cm^−2^), 0.207 µJ pulse^−1^ (9.42 µJ cm^−2^), and 0.212 µJ pulse^−1^ (9.67 µJ cm^−2^) for DPHS, DF, and DCF3 hosts, respectively. In line with PLQE measurements, these values are a fourfold lower than that of pristine F8BT. Furthermore, these results confirm that fluorination, that is, the tuning of the molecular orbitals of the hosts, has a negligible impact on ASE in the blends. In light of these positive results, we carried out a detailed characterization of the optical gain properties in DF/F8BT blends.

Figure 3b shows the ASE threshold energy densities upon 355 nm (*E*_th_^355^) (circles) and 450 nm (*E*_th_^450^) (diamonds) photoexcitation and the ASE peak position as a function of F8BT concentration. The *E*_th_^450^ value of pristine F8BT was 1.3 µJ pulse^−1^ (46 µJ cm^−2^), whereas no ASE was observed upon 355 nm excitation, even for pulse energies up to 2 µJ pulse^−1^ (91 µJ cm^−2^). In DF/F8BT blends, the *E*_th_^450^ values gradually drop for concentrations ranging between 5 and 20 wt%, and subsequently rise at higher concentrations. The lowest *E*_th_^450^ value found was 0.26 µJ pulse^−1^ (9.14 µJ cm^−2^) in 20 wt% blends, which amounts to a fivefold threshold reduction with respect to *E*_th_^450^ value in pristine F8BT film. Notwithstanding, a further ASE threshold reduction is observed in blends upon photoexciting at 355 nm, reaching a 0.112 µJ pulse^−1^ (5.10 µJ cm^−2^) value in blends with 10 wt% F8BT, one order of magnitude lower than the aforementioned value of pristine F8BT film. ASE narrow spectra from F8BT are readily observed in blends with F8BT fraction ranging from 5 to 40 wt%. The FWHM values drop from 17 nm in 5% wt blends to a saturated 11 nm in 40 wt% blends. A progressive redshift of the ASE peak with F8BT doping is observed, as depicted in Figure 3b,c, likely caused by changes in refractive index shifting the fundamental mode. Figure 3d depicts the dependence of ASE peaks and thresholds on film thickness in a 10 wt% F8BT blend. Persisting ASE is seen for film thicknesses ranging from 250 nm (0.26 µJ pulse^−1^ (11.6 µJ cm^−2^) threshold and peak at 556 nm) down to only 115 nm (≈2.24 µJ pulse^−1^ (102 µJ cm^−2^) threshold and peak at 548 nm). We infer that the highly efficient energy transfer between host and guest provides larger optical gain enhancement, in line with previous reports in conjugated polymer blends.^27^, ^36^, ^37^ By increasing the thickness above 150 nm, the ASE threshold is quickly reduced down to the 0.11–0.25 µJ pulse^−1^ range (5–11.5 µJ cm^−2^), while the ASE peak locates at 556 nm. It is well known that the typical thickness of organic layer in a sandwich device is limited by the carrier mobility of organic semiconductors. The possibility of realizing ASE in films as thin as 115 nm opens up prospects for integrating the gain layers in sandwich structure diodes to explore laser action under charge injection.

To further test these blends as laser gain media, we fabricated optically pumped surface emitting distributed feedback (DFB) lasers comprising blend films spin‐coated on grating structures etched into silica substrates. Such structures provide an elegant way of tuning the wavelength of the output emission by altering the supported resonance frequency through control of the blend ratio and/or film thickness. Laser linewidths were around 1 nm FWHM and were limited by the CCD spectrometer resolution. **Figure** **4**a depicts the laser spectra tuning range of the DF/F8BT lasers. We have realized 47 nm tuning from ≈539 to ≈586 nm by using only one grating structure (period Λ = 350 nm, fill factor 50%, etch depth 50 nm) with three different F8BT contents in DF (5% (≈150 nm), 15% (≈150, 165, 190, and 210 nm, respectively), 60% (≈210 nm)). Figure 4b shows the corresponding laser threshold as a function of film thickness for the three blend concentrations. A lowest lasing threshold of ≈2.76 nJ pulse^−1^ (i.e., 10.86 µJ cm^−2^ or 3.62 kW cm^−2^) lasing at ≈562 nm was achieved using 15 wt% F8BT blend as gain media with a film thickness of 165 nm. Figure S8 (Supporting Information) further illustrates how the lowest laser thresholds for different F8BT contents (obtained through film thickness adjustment) remain almost unaltered for a 539–586 nm laser tuning range, confirming that laser tuning is not achieved at the price of increasing the pump fluence.

**Figure 4:**
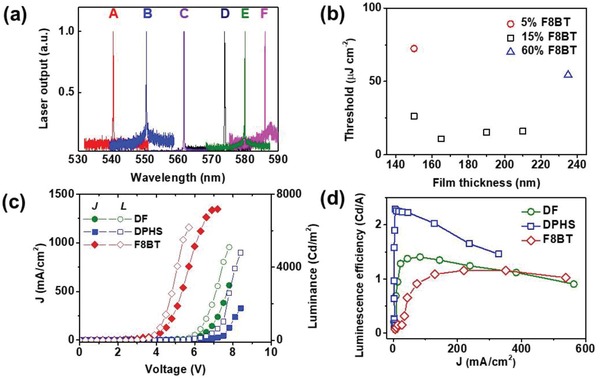
a) Typical laser spectra of F8BT/DF blends on surface‐emitting DFBs (A: 5 wt% F8BT, thickness ≈ 150 nm, peak position 539.2 nm; B: 15% F8BT, thickness ≈ 150 nm, peak position 550.4 nm; C: 15 wt% F8BT, thickness ≈ 165 nm, peak position 561.7 nm; D: 15 wt% F8BT, thickness ≈ 190 nm, peak position 573.9 nm; E: 15 wt% F8BT, thickness ≈ 210 nm, peak position 580 nm; F: 60 wt% F8BT, thickness ≈ 230 nm, peak position 586 nm) obtained upon 355 nm photoexcitation. b) Laser threshold values as a function of the film thickness of F8BT/DF blends: 5 wt% F8BT (circles), 15 wt% F8BT (squares), and 60 wt% F8BT (up‐triangles). c) Current density–voltage–luminance curves (open symbols represent current density and solid symbols represent luminance) and d) luminescence efficiencies versus current density of OLEDs based on F8BT (diamonds) and 20 wt% F8BT on DF (circles) or DPHS (squares) blends.

Next, we investigate the influence of the host energy level shifts on the performance of OLEDs based on blends of F8BT with the three oligomer hosts. Figure 4c shows the current density–voltage–brightness (*J*–*V*–*B*) curves of OLEDs with active layers based on DF/F8BT and DPHS/F8BT blends with a 20 wt% F8BT content. Figure 4d illustrates the dependence of their luminescence efficiencies on current density. Focusing first on DF/F8BT, a light turn‐on voltage (at a measurable brightness of 2 cd m^−2^) of 5.1 V, a 5097 cd m^−2^ (at 6.6 V) maximum brightness and a maximum luminescence efficiency of 1.41 cd A^−1^ (under 83.4 mA cm^−2^ and 1172 cd m^−2^ conditions) were obtained. Similar corresponding values were found in DPHS/F8BT OLEDs: a 5.4 V turn‐on voltage, 4794 cd m^−2^ (6.3 V) maximum brightness, and a 2.29 cd A^−1^ (5.6 mA cm^−2^ and 128 cd m^−2^) current efficiency. In turn, F8BT OLEDs exhibit 3.7 V, 6174 cd m^−2^ (6 V), and 1.16 cd A^−1^ (220 mA cm^−2^ and 2553 cd m^−2^) turn‐on voltage, maximum brightness, and luminescence efficiency, respectively, which at first glance appear comparable or even outperforming values with respect to the blend‐based OLEDs. The higher turn‐on voltage in blends can be attributed to larger differences between the LUMO energy level of the host and the work function of cathode LiF/Al, introducing larger energy barriers for electron injection. Hitherto, the maximum luminescence efficiencies in DF/F8BT and DPHS/F8BT were achieved at very low current densities. The maximum luminescence efficiency in DPHS/F8BT OLEDs (2.29 cd A^−1^) was achieved at 5.6 mA cm^−2^. Equivalent values under such low current densities amount to 0.14 cd A^−1^ in DF/F8BT and <0.06 cd A^−1^ in pristine F8BT OLEDs, that is, a 16‐ and 38‐fold factor lower than DPHS/F8BT, respectively. Such high overall luminescence efficiency in DPHS/F8BT OLEDs is likely due to a better match between the HOMO energy level of host and the work function of PEDOT, resulting in a more balanced electron/hole charge injection. Giving the similar ASE/laser properties of DPHS/F8BT and DF/F8BT blends we conclude that independent tuning of the host energy levels enables an effective change in the injection properties in host/guest blends without jeopardizing light amplification.

In summary, we have demonstrated a strategy that allows for tuning charge injection in the majority host compound while maintaining unaltered PL and ASE spectra and thresholds of blends. Dispersion of F8BT in π‐conjugated oligomer hosts with different fluorination degree enables to shift by 0.3–0.4 eV the HOMO/LUMO levels without causing detectable changes in the ASE threshold with respect to nonfluorinated hosts. Further investigations show that these blends exhibit extremely low ASE threshold, one order of magnitude lower respect to pristine F8BT, due to an efficient host‐to‐guest Förster resonant energy transfer. DFB lasers with low lasing thresholds were achieved across a broad wavelength range from 539 to 586 nm by controlling the F8BT ratio in the blends. Our approach offers a solution for optimization of charge injection and transport properties without detriment to optical gain. A next scope will focus on optically pumped ASE or laser action under current injection^38^ exploring the possibilities of realizing electrically pumped organic lasers based on these blend gain media.

## Experimental Section

Stock solutions of the materials in chloroform (15 mg mL^−1^ for F8BT, 25 mg mL^−1^ for the blue‐emitting oligomer hosts) were first obtained and subsequently mixed in the required ratios to obtain various blend solutions with different weight percentage of F8BT in hosts (1–60 wt% F8BT). Thin films for optical characterization (absorption, PL, PLQE) were prepared by spin‐coating (at speeds ranging from 1000 to 3500 rpm) blend solutions onto precleaned silica (Spectrosil B) substrates leading to films with 180–210 nm thicknesses.

UV–vis absorption and PL spectra were recorded using a Shimadzu UV‐3150 and a Shimadzu RF‐5300 PC spectrophotometer, respectively. PL lifetimes were measured with an Edinburgh FLSP920 fluorescence spectrometer equipped with a 375 nm pulsed laser (55 ps, 20 MHz repetition rate) and a time‐correlated single‐photon counting system. The PLQE values of blend films were measured using the same Edinburgh FLSP920 fluorescence spectrometer with an integrated sphere. For ASE and laser measurements, samples were optically pumped with a Q‐switch neodymium ion doped yttrium aluminium garnate [Nd3+:YAG] laser source which pumped a type‐II β‐BaB_2_O_4_ [BBO] optical parametric oscillator, producing 5 ns pulses in the visible range and 3 ns in the UV at 10 Hz repetition rate. Calibrated neutral density filters were inserted into the beam path to adjust the pulse energy impinging on the sample. For ASE measurements, an adjustable slit and a cylindrical lens were combined to create a narrow excitation stripe (550 µm × 4 mm for λ = 355 nm excitation and 700 µm × 4 mm for λ = 450 nm) placed at the edge of sample film. The edge emission from samples was collected with an optical fiber and sent onto a spectrometer (Andor, Shamrock 500) equipped with a CCD detector (Newton 940). At sufficient excitation intensities, the spontaneously emitted photons that are waveguided along the stripe‐shaped gain region are amplified via stimulated emission. This process results in most of the light being emitted from the ends of the stripe. Here, we define *E*_th_^ASE^ as the incident fluence at which the FWHM linewidth falls to half way between the linewidths of the PL and ASE spectra. The film thickness for ASE measurements was around 200 nm. The energy of the photoexciting pulse was determined with an energy/power meter. DFB lasers were optically pumped with the same source and detected using the same detector. DFB lasers were fabricated by prepatterning silica substrates with 1D surface grating structures (grating period Λ = 350 nm, fill factor 50%, etch depth 50 nm) and then spin‐coating thin films on top. The pump excitation area employed for monitoring laser emission was a circular spot with 180 µm in diameter (2.54 × 10^−4^ cm^2^ in area). All thickness measurements were obtained with a DektakXT (Bruker). AFM measurements were obtained using a JPK Nanowizard II coupled to a Ti‐U inverted optical microscope.

OLED devices with a ITO/PEDOT(25 nm)/polymer(70 nm)/LiF(1 nm)/Al(150 nm) architecture were fabricated. The schematic energy level diagram of each layer in the structure is shown in Figure S9 (Supporting Information). Prepatterned ITO glass substrates (purchased from Wan Qing Ltd.) were cleaned with soap (Hellmanex) and rinsed with deionized water. Then, the substrates were sonicated in a mixture of acetone:isopropanol (1:1 v/v), rinsed with ethanol and dried with compressed nitrogen. After that, the ITO glasses were treated with UV–ozone for 15 min, following by instant spin‐coating of PEDOT:PSS on top. PEDOT‐coated substrates were then transferred onto a hot plate (120 °C) to remove the residual moisture. Blend solutions (DF/F8BT and DPHS/F8BT with 20% F8BT content, 10 mg mL^−1^ in chloroform) and pristine F8BT (8 mg mL^−1^ in toluene) were then deposited on top by spin‐coating at a 2000 rpm spin speed. The thickness of resulted active layers was controlled to be around 70 nm. The devices were then transferred into the thermal evaporator for a subsequent deposition of LiF interlayer and an aluminum cathode at a basic pressure of 10^−4^Pa. The active emission area was defined by a shadow mask is 0.1 cm^2^. The ensuing performances of encapsulated devices were tested in a N_2_ atmosphere dry box (oxygen, water < 1 ppm). EL spectra were collected by an optical fiber attached to the Ocean Optics HR 4000 spectrometer. The current density–operation voltage–luminance (*J*–*V*–*L*) were collected by a Keithley 2400 source meter and a calibrated silicon photodiode.

## Conflict of Interest

The authors declare no conflict of interest.

## Supporting information

Supplementary

## References

[1] N. Tessler , G. J. Denton , R. H. Friend , Nature 1996, 382, 695.

[2] W. Holzer , A. Penzkofer , S. H. Gong , A. P. Davey , W. J. Blau , Opt. Quantum Electron. 1997, 29, 713.

[3] I. D. W. Samuel , G. A. Turnbull , Chem. Rev. 2007, 107, 1272. 17385928 10.1021/cr050152i

[4] C. Sun , M. M. Mróz , J. R. Castro Smirnov , L. Lüer , D. Hermida‐Merino , C. Zhao , M. Takeuchi , K. Sugiyasu , J. Cabanillas‐González , J. Mater. Chem. C 2018, 6, 6591.

[5] M. Karl , J. M. E. Glackin , M. Schubert , N. M. Kronenberg , G. A. Turnbull , I. D. W. Samuel , M. C. Gather , Nat. Commun. 2018, 9, 1525. 29717120 10.1038/s41467-018-03874-w PMC5931618

[6] Y. Xu , G. Hai , H. Xu , H. Zhang , Z. Zuo , Q. Zhang , R. Xia , C. Sun , J. Castro‐Smirnov , A. Sousaraei , S. Casado , M. R. Osorio , D. Granados , I. Rodriguez , J. Cabanillas‐Gonzalez , Adv. Opt. Mater. 2018, 6, 1800263.

[7] D.‐H. Kim , A. D'Aléo , X.‐K. Chen , A. D. S. Sandanayaka , D. Yao , L. Zhao , T. Komino , E. Zaborova , G. Canard , Y. Tsuchiya , E. Choi , J. W. Wu , F. Fages , J.‐L. Brédas , J.‐C. Ribierre , C. Adachi , Nat. Photonics 2018, 12, 98.

[8] A. J. C. Kuehne , M. C. Gather , Chem. Rev. 2016, 116, 12823. 27501192 10.1021/acs.chemrev.6b00172

[9] A. S. D. Sandanayaka , T. Matsushima , F. Bencheikh , K. Yoshida , M. Inoue , T. Fujihara , K. Goushi , J.‐C. Ribierre , C. Adachi , Sci. Adv. 2017, 3, e1602570. 28508042 10.1126/sciadv.1602570 PMC5409494

[10] D. Pile , S. Forrest , Nat. Photonics 2010, 4, 402.

[11] S. Kéna‐Cohen , S. R. Forrest , Nat. Photonics 2010, 4, 371.

[12] Y. Hu , W. Cai , L. Ying , D. Chen , X. Yang , X.‐F. Jiang , S. Su , F. Huang , Y. Cao , J. Mater. Chem. C 2018, 6, 2690.

[13] S. Liu , C. Zhong , S. Dong , J. Zhang , X. Huang , C. Zhou , J. Lu , L. Ying , L. Wang , F. Huang , Y. Cao , Org. Electron. 2014, 15, 850.

[14] G. Heliotis , R. D. Xia , G. A. Turnbull , P. Andrew , W. L. Barnes , I. D. W. Samuel , D. D. C. Bradley , Adv. Funct. Mater. 2004, 14, 91.

[15] R. Xia , G. Heliotis , P. N. Stavrinou , D. D. C. Bradley , Appl. Phys. Lett. 2005, 87, 031104.

[16] R. D. Xia , G. Heliotis , D. D. C. Bradley , Appl. Phys. Lett. 2003, 82, 3599.

[17] G. Heliotis , R. Xia , D. D. C. Bradley , G. A. Turnbull , I. D. W. Samuel , P. Andrew , W. L. Barnes , Appl. Phys. Lett. 2003, 83, 2118.

[18] A. Gadisa , W. Mammo , L. M. Andersson , S. Admassie , F. Zhang , M. R. Andersson , O. Inganas , Adv. Funct. Mater. 2007, 17, 3836.

[19] A. C. Morteani , A. S. Dhoot , J.‐S. Kim , C. Silva , N. C. Greenham , C. Murphy , E. Moons , S. Cina , J. H. Burroughes , R. H. Friend , Adv. Mater. 2003, 15, 1708.

[20] F. Babudri , G. M. Farinola , F. Naso , R. Ragni , Chem. Commun. 2007, 38, 1003. 10.1039/b611336b 17325792

[21] S. Shao , J. Ding , L. Wang , X. Jing , F. Wang , J. Am. Chem. Soc. 2012, 134, 15189. 22950598 10.1021/ja305634j

[22] W. Lu , J. Kuwabara , T. Iijima , H. Higashimura , H. Hayashi , T. Kanbara , Macromolecules 2012, 45, 4128.

[23] T. Zhang , R. Wang , H. Ren , Z. Chen , J. Li , Polymer 2012, 53, 1529.

[24] Q. Zhang , Y. Zhang , W. Xu , X. Li , J. Liu , X. Guo , R. Xia , W. Huang , Opt. Express 2015, 23, A465. 26072871 10.1364/OE.23.00A465

[25] Q. Niu , Q. Zhang , W. Xu , Y. Jiang , R. Xia , D. D. C. Bradley , D. Li , X. Wen , Org. Electron. 2015, 18, 95.

[26] R. Xia , G. Heliotis , Y. B. Hou , D. D. C. Bradley , Org. Electron. 2003, 4, 165.

[27] Q. Zhang , J. Liu , Q. Wei , X. Guo , Y. Xu , R. Xia , L. Xie , Y. Qian , C. Sun , L. Lüer , J. Cabanillas‐Gonzalez , D. D. C. Bradley , W. Huang , Adv. Funct. Mater. 2018, 28, 1705824.

[28] A. Charas , A. L. Mendonca , J. Clark , J. Cabanillas‐Gonzalez , L. Bazzana , A. Nocivelli , G. Lanzani , J. Morgado , Front. Optoelectron. China 2010, 3, 45.

[29] M. A. Stevens , C. Silva , D. M. Russell , R. H. Friend , Phys. Rev. B 2001, 63, 165213.

[30] W. Lee , H. D. Lee , J. H. Bae , J. W. Jung , Org. Electron. 2016, 39, 85.

[31] Z. Chen , W. Zhang , J. Huang , D. Gao , C. Wei , Z. Lin , L. Wang , G. Yu , Macromolecules 2017, 50, 6098.

[32] Y. Qian , Q. Wei , G. Del Pozo , M. M. Mroz , L. Lüer , S. Casado , J. Cabanillas‐Gonzalez , Q. Zhang , L. Xie , R. Xia , W. Huang , Adv. Mater. 2014, 26, 2937. 24665075 10.1002/adma.201305355

[33] B. Dänekamp , B. Kobin , S. Bhattacharyya , S. Hecht , B. Milián‐Medina , J. Gierschner , Phys. Chem. Chem. Phys. 2016, 18, 16501. 27263988 10.1039/c6cp02045c

[34] B. Milián‐Medina , J. Gierschner , J. Phys. Chem. Lett. 2017, 8, 91. 27958747 10.1021/acs.jpclett.6b02495

[35] A. J. Cadby , R. Dean , C. Elliott , R. A. L. Jones , A. M. Fox , D. G. Lidzey , Adv. Mater. 2007, 19, 107.

[36] Z. Yu , X. Guo , Q. Zhang , L. Chi , T. Chen , R. Xia , L. Wu , L. Lüer , J. Cabanillas‐Gonzalez , J. Phys. Chem. C 2016, 120, 11350.

[37] R. Xia , P. N. Stavrinou , D. D. C. Bradley , Y. Kim , J. Appl. Phys. 2012, 111, 123107.

[38] B. H. Wallikewitz , M. de la Rosa , J. H. W. M. Kremer , D. Hertel , K. Meerholz , Adv. Mater. 2010, 22, 531. 20217748 10.1002/adma.200902451

