# Supplementary material for: Concurrent Optical Gain Optimization and Electrical Tuning in Novel Oligomer:Polymer Blends with Yellow‐Green Laser Emission
Source: Adv Sci (Weinh). 2018 Nov 8;6(1):1801455. doi: 10.1002/advs.201801455 (PMC6325601; doi:10.1002/advs.201801455)
Supplement: Supplementary file 1 — Supplementary [file ADVS-6-1801455-s001.pdf]

Copyright WILEY-VCH Verlag GmbH & Co. KGaA, 69469 Weinheim, Germany, 2018.

ADVANCED SCIENCE
Open Access

Supporting Information

for *Adv. Sci.,* DOI: 10.1002/advs.201801455

Concurrent Optical Gain Optimization and Electrical Tuning in Novel Oligomer:Polymer Blends with Yellow-Green Laser Emission

Qi Zhang, Qi Wei, Xiangru Guo, Gang Hai, Huizhi Sun, Jiewei Li, Ruidong Xia, Yan Qian,* Santiago Casado, José Raúl Castro-Smirnov, and Juan Cabanillas-Gonzalez**

Copyright WILEY-VCH Verlag GmbH & Co. KGaA, 69469 Weinheim, Germany, 2016.

Supporting Information

Concurrent optical gain optimization and electrical tuning in novel oligomer:polymer blends with yellow-green laser emission

Qi Zhang,[#] Qi Wei , Xiangru Guo, Gang Hai, Huizhi Sun, Jiewei Li, Ruidong Xia,* Yan Qian,* Santiago Casado, José Raúl Castro-Smirnov, Juan Cabanillas-Gonzalez*

Materials

Boron fluoride ethyl ether were purchased from SHANGHAILINGFENG Chemical CO. Tetrakis(triphenylphosphine)palladium(0), 2,7-dibromo-9H-fluoren-9-one, (3,5-difluorophenyl)boronic acid, (4-(trifluoromethyl)phenyl)boronic acid and [1,1'-biphenyl]-4-ylboronic acid were purchased from Aldrich Chemical Co. 2-bromo-9,9-dioctyl-9H-fluorene and 9-octyl-9H-carbazole were obtained according to our previous literatures.

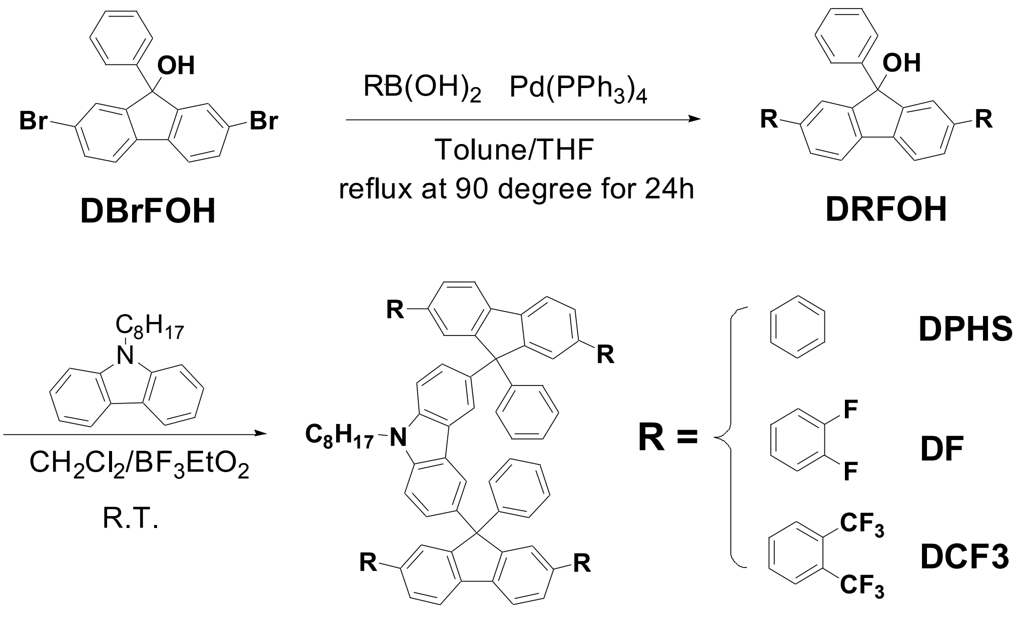

Scheme 1. The synthetic routes of DPHS, DF and DCF3.

Synthesis of 9-octyl-3,6-bis(2,7,9-triphenyl-9H-fluoren-9-yl)-9H-carbazole (DPHS)

$BF3{\cdot}Et_2O$ (1.3 ml, 9.16 mmol) was added to 100ml anhydrous CH_2Cl_2 solution of **DRFOH**(1.0 g, 2.4 mmol) and 9-octyl-9H-carbazole (0.62 g, 2.2 mmol), and the batch was stirred over a period of 5 h at room temperature. The resulting mixture was washed by water and extracted with CH_2Cl_2. The residue then subjected to silica-gel column chromatography to afford 0.4g white solid of DPhS in 24% yield.1H NMR (400 MHz, $CDCl_3$) δ 7.87 (s, 1H), 7.84 (d, J = 7.9 Hz, 4H), 7.64 (s, 4H), 7.58 (d, J = 7.9 Hz, 4H), 7.51 (d, J = 7.5 Hz, 8H), 7.39-7.26 (m, 18H), 7.20-7.18 (m, 8H), 4.15 (t, J = 7.1 Hz, 2H) 1.81-1.71 (2, 1H), 1.38-1.17 (m, 10H), 0.81 (t, J = 7.0 Hz, 3H).13C NMR (101 MHz, $CDCl_3$) δ 153.05, 146.47, 141.39, 140.75, 139.67, 138.97, 135.94, 128.66, 128.29, 127.20, 127.09, 126.66, 126.45, 125.10, 122.51, 120.44, 119.80, 108.61, 65.77, 63.20, 43.20, 34.47, 31.77, 30.87, 29.72, 29.46, 29.30, 29.21, 29.12, 29.03, 28.61, 27.29, 26.74, 22.56, 19.94, 14.05.MALDI-TOF m/z:1062.82

[M].Anal.Calcd for $C_{82}H_{65}N$ (1063.51): C, 92.53; H, 6.16; N, 1.32. Found: C, 92.93; H, 5.96; N, 1.44.

Synthesis of 3,6-bis(2,7-bis(3,5-difluorophenyl)-9-phenyl-9H-fluoren-9-yl)-9-octyl-9H-carbazole (DF)

$BF_3 \cdot Et_2O$ (1.3 ml, 9.16 mmol) was added to 100ml anhydrous CH_2Cl_2 solution of **DRFOH** (1.0 g, 2.1 mmol) and 9-octyl-9H-carbazole (0.53 g, 1.9 mmol), and the batch was stirred over a period of 5 h at room temperature. The resulting mixture was washed by water and extracted with CH_2Cl_2. The residue then subjected to silica-gel column chromatography to afford 0.73g (48%) white solid of DF. 1H NMR (400 MHz, $CDCl_3$) δ 7.85(d, J= 7.9 Hz, 4H), 7.73 (s, 2H), 7.56 (s, 4H), 7.53(d J= 7.9, 4H), 7.34-7.21(m, 14H), 6.99(d, J= 8.5, 8H), 6.76 (t, J= 8.8, 4H), 4.18(t, J= 7.3, 2H), 1.83-1.76 (m, 2H), 1.37-1.20 (m, 10H), 0.82 (t, J= 8.7, 3H).13C NMR (101 MHz, CDC13) δ 164.52, 164.39, 162.05, 161.92, 153.31, 145.86, 144.66, 144.57, 144.47, 139.75, 139.72, 138.72, 135.42, 128.49, 128.11, 126.97, 126.67, 126.49, 124.90, 122.37, 120.92, 119.35, 110.09, 110.02, 109.90, 109.83, 108.95, 102.64, 102.39, 102.14, 65.78, 43.12, 31.74, 29.28, 29.13, 29.05, 27.28, 22.54, 14.02.MALDI-TOF m/z: 1207.02 [M].Anal.Calcd for $C_{82}H_{57}F_8N$ (1207.44): C, 81.51; H, 4.75; N, 1.16. Found: C, 81.73; H, 4.92; N, 1.23.

Synthesis of 3,6-bis(2,7-bis(3,5-bis(trifluoromethyl)phenyl)-9-phenyl-9H-fluoren-9-yl)-9-octyl-9H-carbazole (DCF3)

$BF_3 \cdot Et_2O$ (1.3 ml, 9.16 mmol) was added to 100ml anhydrous CH_2Cl_2 solution of DRFOH (0.9 g, 1.4 mmol) and 9-octyl-9H-carbazole (0.37 g, 1.3 mmol), and the batch was stirred over a period of 5 h at room temperature. The resulting mixture was washed by water and extracted with CH_2Cl_2. The residue then subjected to silica-gel column chromatography to afford 0.57g white solid of DCF_3(**7**) in 53% yield.^{1}H NMR (400 MHz, $CDCl_3$) δ 7.93 (d, J= 8.0 Hz, 4H), 7.87 (s, 8H), 7.81 (s, 6H), 7.61-7.59 (d, J= 7.9, 4H), 7.55 (s, 4H), 7.35-7.33 (m, 4H), 7.26 - 7.24 (m, 10H), 4.16 (t, 7.3Hz, 2H) , 1.80-1.73 (m, 2H), 1.32-1.18 (m, 10H), 0.80(t, J= 6.8 Hz, 3H).
^{13}C NMR (101 MHz, $CDCl_3$) δ 153.90, 145.41, 143.26, 139.89, 139.86, 138.33, 135.15, 132.52, 132.19, 131.86, 131.53, 128.64, 128.05, 127.37, 127.22, 127.07, 126.39, 125.00, 124.67, 122.52, 121.96, 121.32, 120.85, 119.56, 108.98, 66.07, 43.19, 31.70, 29.72, 29.25, 29.06, 28.98, 27.25, 22.49, 13.96.MALDI-TOF m/z: 1607.44 [M].Anal.Calcd for $C_{90}H_{57}F_{24}N$ (1607.41): C, 67.21; H, 3.57; N, 0.87. Found: C, 67.33; H, 3.42; N, 0.98.

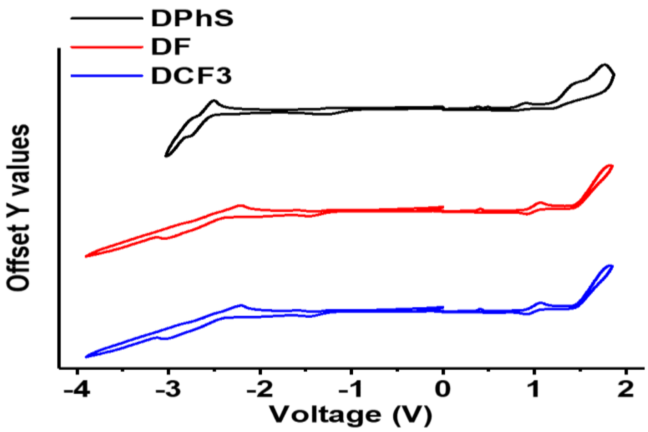

Figure S1. Oxidative and reductive cyclic voltammetry plots of DPHS, DF, DCF3 measured in CH_2Cl_2 and THF solutions, respectively, with Bu_4NPF_6 as the electrolyte and ferrocene/ferrocenium (Fe/Fe^+) couple as internal reference.

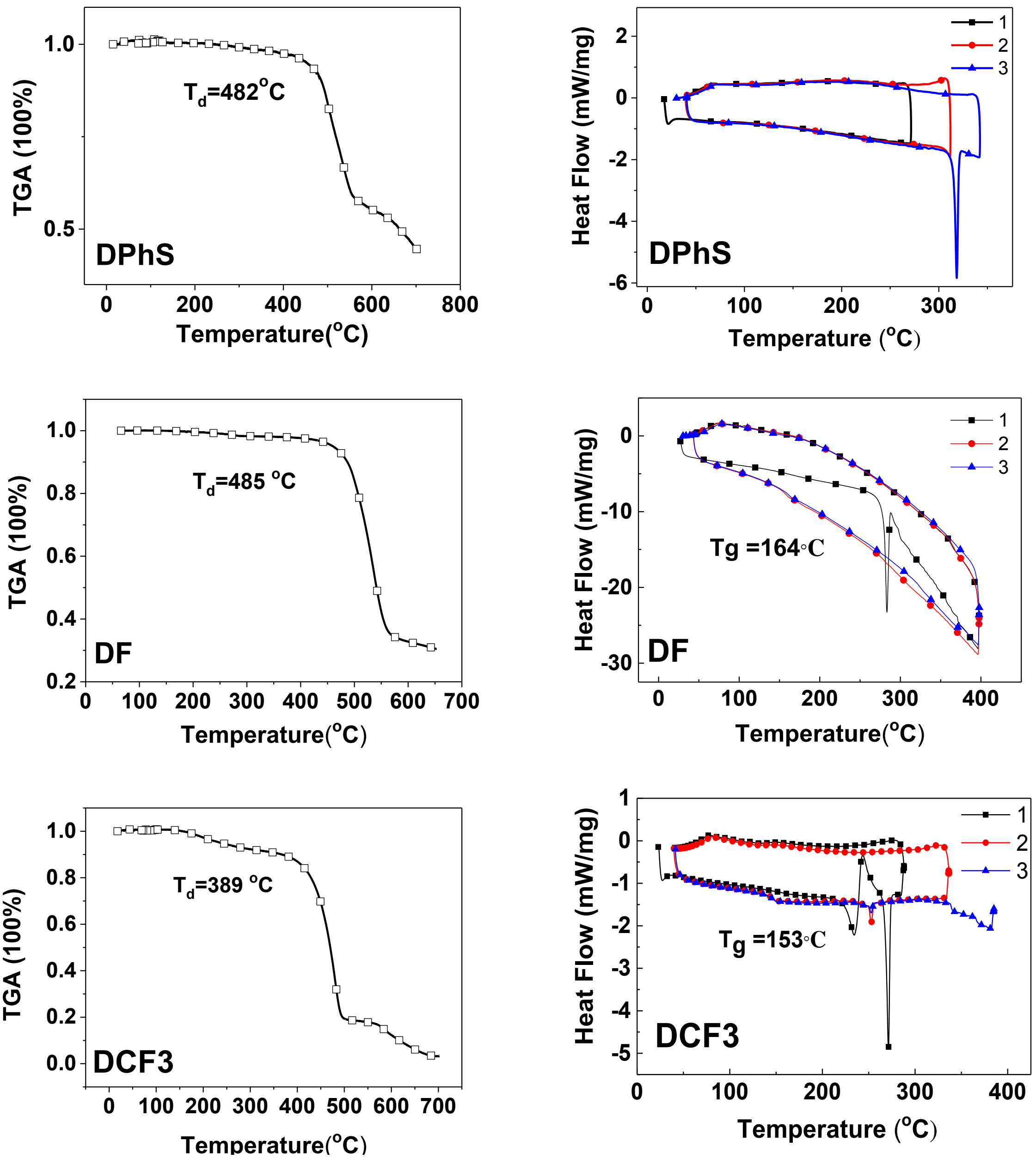

Figure S2. The TGA (left) and DSC (right) curves of DPHS, DF, DCF3 (black line 1, red line 2 and blue line 3 are the first, second and third cycle, respectively).

	HOMO	LUMO	HOMO c./m. (eV)	LUMO c./m. (eV)
**DPHS**	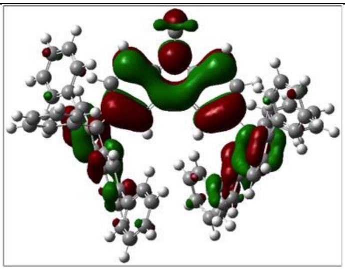	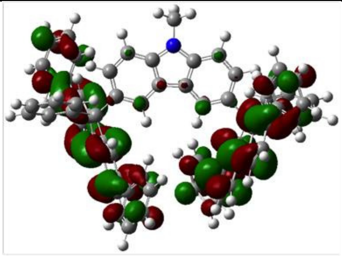	**-5.220/ -5.46**	**-1.207/ -2.16**
**DF**	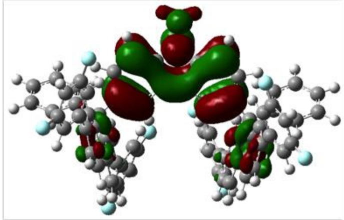	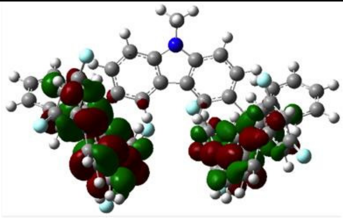	**-5.427/ -5.65**	**-1.624/ -2.45**
**DCF3**	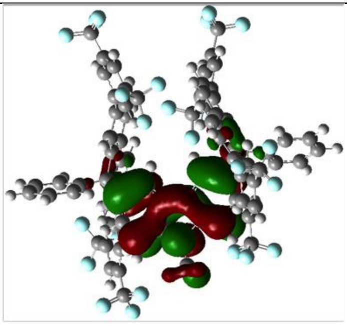	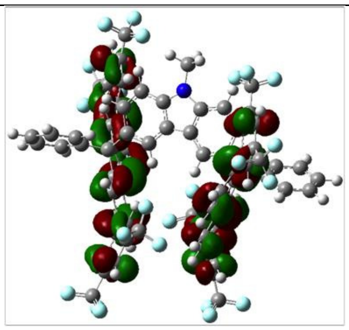	**-5.624/ -5.66**	**-1.911/ -2.51**

Figure S3. The HOMO/LUMO molecular orbitals and energy levels of the oligomer hosts of DPHS, DF and DCF3, calculated by B3LYP/6-31G(D). The c./m. in the table represents calculated and measured HOMO/LUMO values respectively.

Table S1. Key parameters of the host oligomers

Compounds	τ (ns)	η(%)	E_{HOMO}	E_{LUMO}	Eg	Max abs.	Max PL
DPHS	2.94	40	-2.16	-5.46	3.3	309	395
DF	9.00	51	-2.45	-5.65	3.20	308	403
DCF_3	11.55	55	-2.51	-5.66	3.36	307	414

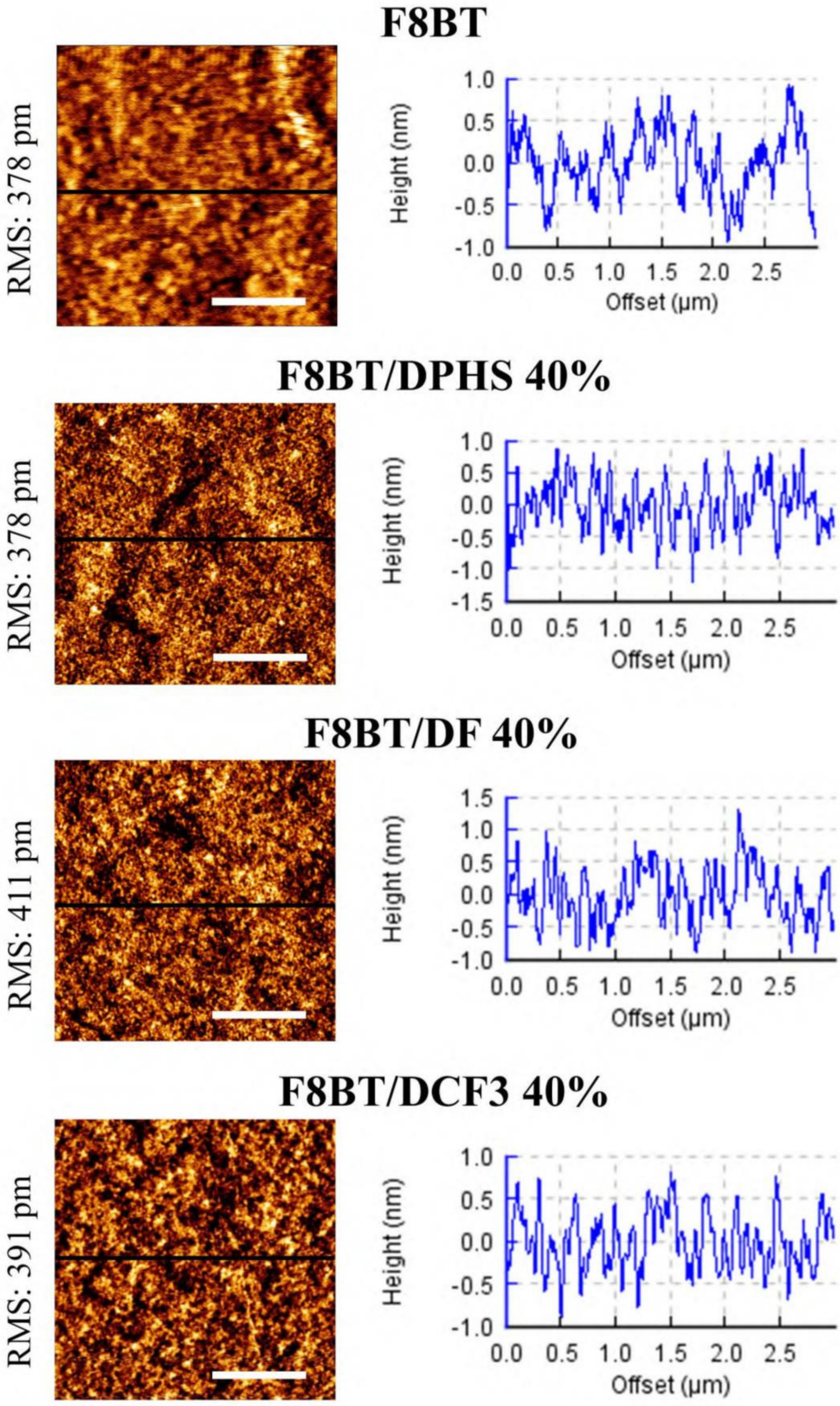

Figure S4. (Left) AFM images of pristine F8BT and blends films. The white scale bar in the AFM images is 1 μm. Profiles obtained across a scanned line. RMS values are provided on the Y-axis of AFM images.

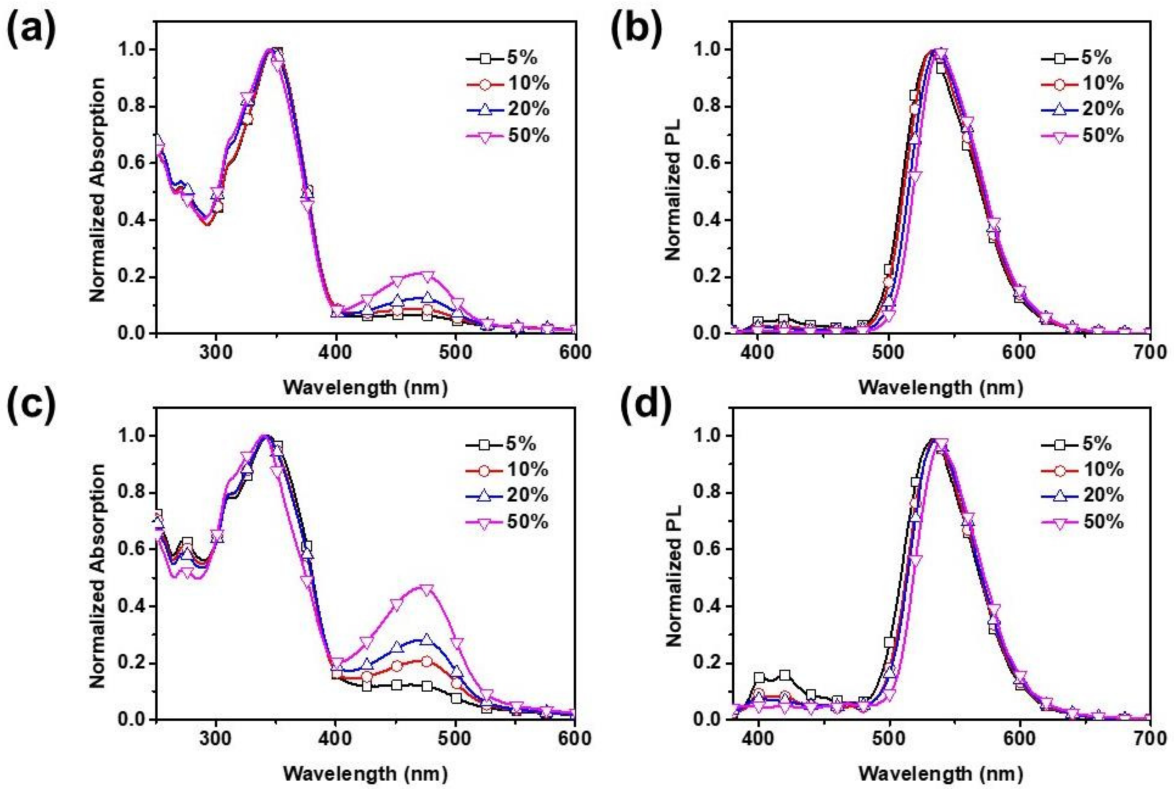

Figure S5 Absorption and PL spectra of F8BT/DPHS (a), (b) and F8BT/DCF3 (c), (d) blend films, respectively, with different F8BT contents: 5 wt.% (squares), 10 wt.% (circles), 20 wt.% (up-triangles) and 50 wt.% (down-triangles). The PL spectra were obtained upon 355 nm photoexcitation.

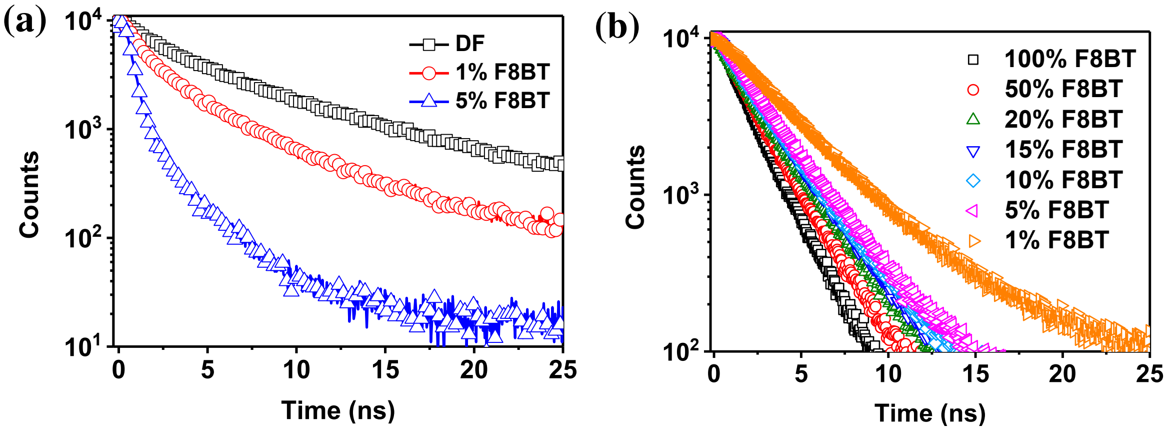

Figure S6. (a) PL lifetime measurements detecting at 420 nm (DF emission) of pristine DF (squares), 1 wt.% F8BT (circles) and 5 wt.% F8BT (triangles) blends. (b) PL lifetime measurements detecting at 560 nm (F8BT emission) in F8BT/DF blends with different F8BT concentration. The PL decay curves were in all cases fitted with single or double exponential curves and averaged lifetimes were obtained. All samples were excited at 375 nm with a picosecond laser as described in experimental section. The data is summarized in Table S2.

Energy transfer rate in the blend film between the host and guest can be estimate as followed:

$k_{host}= k_{host\ r}+k_{host\ nr} =1/\ \tau$

Φ_{host}= $k_{host\ r}$ /($k_{host\ r}$+$k_{host\ nr}$)
Φ_{blend}= $k_{host\ r}$ /($k_{host\ r}$+$k_{host\ nr}$+k_{ET})

In DF film, $k_{host\ r}$=5.6 x 10^7 s^{-1}
In 1 wt.% F8BT blend film, k_{ET}=2.9 x 10^8 s^{-1}.
In 5 wt.% F8BT blend film, k_{ET}=1.36 x 10^9 s^{-1}.

Table S2. PL lifetime of F8BT/DF blend films at the wavelength of 560 nm

F8BT fraction (%)	100	50	20	15	10	5	1
τ (ns)	1.88	2.07	2.39	2.42	2.52	2.70	4.14

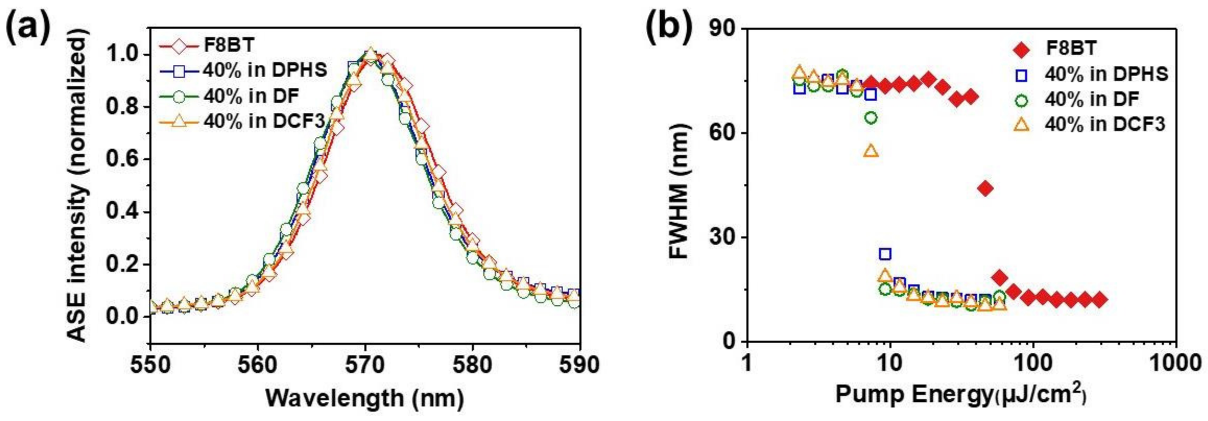

Figure S7. (a) Comparison of ASE spectra and (b) FWHM as a function of pump energy for 40 wt. % F8BT films blended with different host materials (photoexcited at 355 nm) and for pristine F8BT (photoexcited at 450 nm).

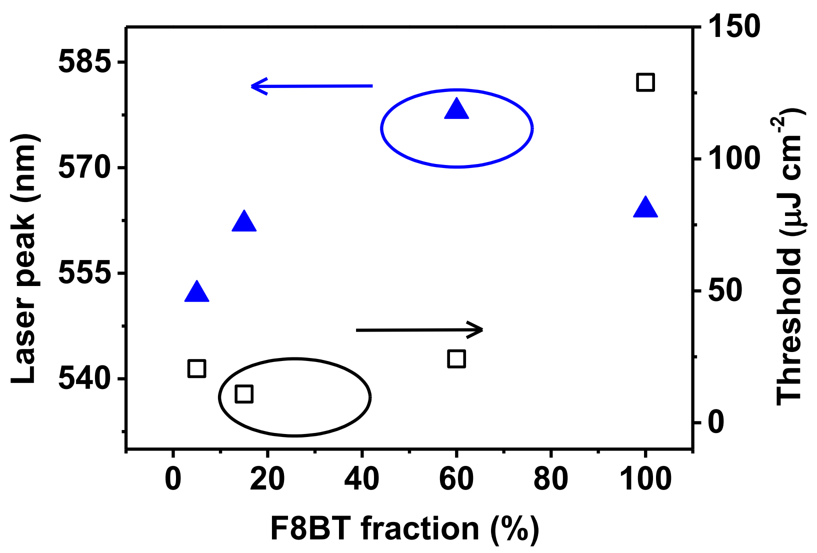

Figure S8. The lowest laser threshold and correlated wavelength as a function of F8BT fraction. (Film thickness: 5 wt.% 190 nm, 15 wt.% 165 nm, 60 wt.% 180 nm, 100 wt.% 160 nm).

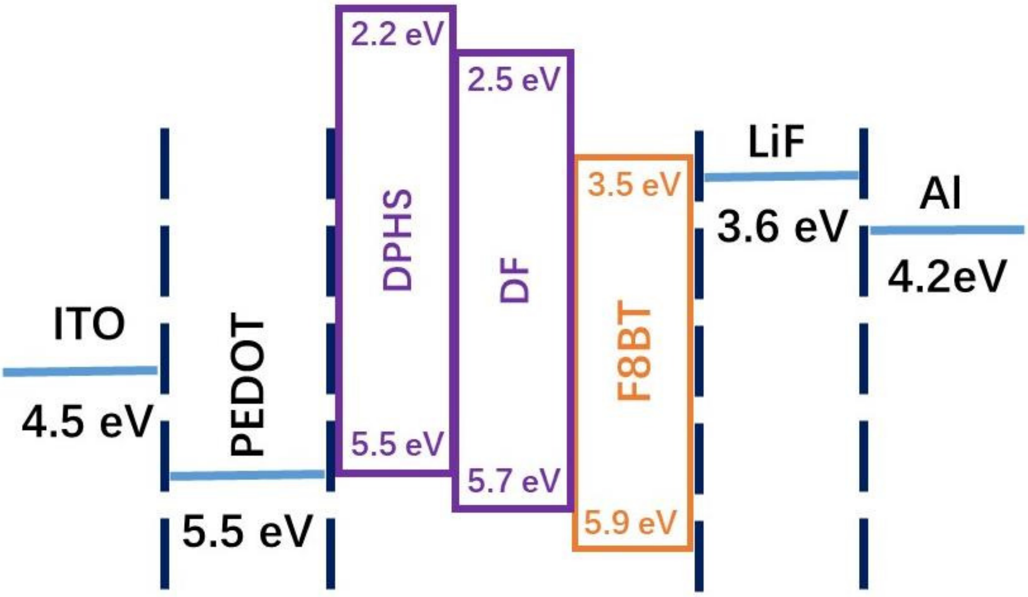

Figure S9. The energy level schematic diagram of each layer in the device.
